# Utilization of Clarified Butter Sediment Waste as a Feedstock for Cost-Effective Production of Biodiesel

**DOI:** 10.3390/foods8070234

**Published:** 2019-06-29

**Authors:** Alok Patel, Km Sartaj, Parul A. Pruthi, Vikas Pruthi, Leonidas Matsakas

**Affiliations:** 1 Biochemical Process Engineering, Division of Chemical Engineering, Department of Civil, Environmental and Natural Resources Engineering, Luleå University of Technology, 971 87 Luleå, Sweden; 2 Molecular Microbiology Laboratory, Biotechnology Department, Indian Institute of Technology Roorkee (IIT-R), Roorkee 247667, India

**Keywords:** clarified butter sediment waste, hydrophobic substrates, oleaginous yeast, lipids, biodiesel, fatty acid methyl esters

## Abstract

The rising demand and cost of fossil fuels (diesel and gasoline), together with the need for sustainable, alternative, and renewable energy sources have increased the interest for biomass-based fuels such as biodiesel. Among renewable sources of biofuels, biodiesel is particularly attractive as it can be used in conventional diesel engines without any modification. Oleaginous yeasts are excellent oil producers that can grow easily on various types of hydrophilic and hydrophobic waste streams that are used as feedstock for single cell oils and subsequently biodiesel production. In this study, cultivation of *Rhodosporidium kratochvilovae* on a hydrophobic waste (clarified butter sediment waste medium (CBM)) resulted in considerably high lipid accumulation (70.74% w/w). Maximum cell dry weight and total lipid production were 15.52 g/L and 10.98 g/L, respectively, following cultivation in CBM for 144 h. Neutral lipids were found to accumulate in the lipid bodies of cells, as visualized by BODIPY staining and fluorescence microscopy. Cells grown in CBM showed large and dispersed lipid droplets in the intracellular compartment. The fatty acid profile of biodiesel obtained after transesterification was analyzed by gas chromatography-mass spectrometry (GC–MS), while its quality was determined to comply with ASTM 6751 and EN 14214 international standards. Hence, clarified sediment waste can be exploited as a cost-effective renewable feedstock for biodiesel production.

## 1. Introduction

Current research seeks to find sustainable solutions to three eminent problems, namely global warming, energy crisis, and waste generation [1]. This implies increased attention towards biomass-based biofuels [2,3]. Several renewable transportation fuels already exist in the market; their aim being to diminish greenhouse gas emissions and reliance on petroleum-based fuels [4]. They include ethanol from corn and sugarcane, as well as biodiesel from vegetable crops, such as soy, palm, and rapeseed. However, the rising demand for transportation fuels, and the food versus fuel debate raises questions about their sustainability [5], sparking the need for alternative sources. Some non-edible feedstocks, such as waste cooking oils [6,7] and Soursop seed oil [8] are extensively used for biodiesel production but due to the presence of impurities, water, and high amount of free fatty acids, these substrates need extra pretreatments [7] or some modifications in transesterification reactions, such as a two-step process [8]. Most importantly, until conventional vehicle engines are replaced by electrical-powered ones, liquid biofuels remain the only viable renewable and sustainable fuel source for the transportation sector [9]. The vast amounts of liquid fuel required for transportation, create an important incentive towards increased production of renewable liquid fuels. Among the various alternatives, such as ethanol, biogas, and biodiesel, the latter holds an advantage as it can be directly used in conventional diesel engines without any modifications [5]. Chemically, biodiesel is a mixture of fatty acid methyl esters (FAMEs) and can be produced by using any type of fatty acid, regardless of its origin [10]. Oleaginous microorganisms, capable of accumulating high amounts of intracellular lipids, offer a sustainable solution to the use of edible oils for biodiesel production. Such microorganisms can synthesize lipids in the form of droplets [11] and some of them can accumulate more than 60% of lipids in their cellular compartments, under optimized cultivation conditions [12].

According to the Organization of Petroleum Exporting Countries, global oil demand will rise to 109.4 million barrels per day, of which diesel alone is expected to increase by 5.7 million barrels per day by 2040 [13]. As a consequence, the need to replace fossil fuels with renewable sources means that demand for biodiesel will follow a similar trend. To meet this challenge, a low-cost production process should be established. However, a prominent issue in biofuel production is cost, particularly the high cost of feedstock (60–75% of the total cost when using oleaginous microorganisms) [14]. To reduce production costs, novel strains capable of microbial lipids production using cheap waste streams, such as prickly-pear juice, industrial fats, monosodium glutamate wastewater, and crude glycerol derived from yellow grease, have been introduced [15,16,17].

Among the various types of microorganisms (microalgae, fungi, bacteria, and yeasts), the latter has been extensively studied due to their ability to grow on different types of waste substrates obtained from industrial or agricultural processes [3]. They utilize preferably hydrophilic substrates, and synthesize lipids in their cytoplasmic compartment via the de novo pathway under nutrient-limited conditions [18]. In contrast, only yeasts capable of synthesizing lipids via the ex novo pathway can utilize hydrophobic substrates. *Yarrowia lipolytica* is one of the most studied oleaginous yeasts for ex novo lipid accumulation [19], together with *Cryptococcus*, *Rhodosporidium*, *Geotrichum*, and *Trichosporon*, which can all synthesize lipids from hydrophobic substrates. Several multigene families have been identified in these oleaginous yeasts that take part in the degradation of hydrophobic substrates at the cell surface and their assimilation into triacylglycerol (TAG) in the cellular compartment [20]. Uptake of hydrophobic substrates is started by extracellular lipases, which degrade such substrates into free fatty acids (FFA), prior to their internalization by active transport or gradient-based diffusion [20,21]. To facilitate FFA internalization or increase contact between the cell’s surface and the substrate, oleaginous yeasts’ surfaces are modified with protrusions [22,23]. After internalization, FFA are either consumed for cell growth or are taken up by the ex novo lipid synthesis pathway. In the latter case, they are reserved in the form of lipid bodies with same compositional contents or are enzymatically modified to yield fatty acids present in the medium [24]. β-oxidation plays a crucial role after assimilation of FFAs to cover the energy demands for cell growth and repair or synthesis of other metabolites [23]. 

The main hydrophobic substrates used for microbial cultivation are FFA from various sources, such as wastewater from food industries and dairy industries, waste cooking oil, and waste fish oils [20,21,25,26]. Millions of tons of waste cooking oil and animal fats are produced per year; these are responsible for environmental pollution when directly disposed into landfill sites or water bodies. Moreover, there is still no efficient method for the processing of used oils from households [27]. Therefore, utilization of hydrophobic substrates by oleaginous yeasts offers a cost-effective approach for biodiesel production, as well as a solution for managing such detrimental waste [27,28]. 

Ghee or clarified butter is an important global meal ingredient [29]. Clarified butter sediment waste or ghee residue is one of the largest byproducts of the dairy industry, with almost 91,000 tons per annum being produced in India alone [30]. It is generated after coagulation of the solid, non-fat part of the cream of milk, during the preparation of ghee [31]. It can be easily separated from clarified butter by filters, muslin cloth or continuous centrifugal clarifiers. The yield of clarified butter sediment waste depends on the raw materials and the method of preparation [32]. The maximum reported average yield is 12% of the initial raw material through the direct creamery method, followed by 3.7% yield in creamery butter and desi butter methods [33]. The chemical composition of clarified butter sediments waste varies according to the initial raw cream quality and method of preparation; it usually consists of fat (32–70%), protein (12–39%), moisture (8–30%), lactose (2–14%), and ash (1–8%) [29]. The residue also contains a high quantity of phenolic compounds that might be harmful for human consumption [34]. As it is usually discarded as waste into the environment, butter sediments waste could represent a low-cost waste feedstock for biodiesel production and, as such, could be less of a burden for the environment [35]. Previously, ghee residue/clarified butter sediment waste was utilized for the cost-effective production of microbial lipases through solid-state fermentation. For example, *Bacillus subtilis* (NCIM 2063) and *Proteus* spp. produced high amounts of lipases, corresponding to 35.93 U/mg and 41.27 U/mg, respectively, when cultivated on solid ghee residue [31]. Clarified butter sediment waste from the dairy industry has also been used for cultivating bacteria (*Bacillus sphaericus* and *Bacillus thuringiensis* serovar *israelensis*) with biomass yields of 9.7 g/L and at a lower cost than with a conventional nutrient yeast extract salt medium [35]. 

**Figure 1:**
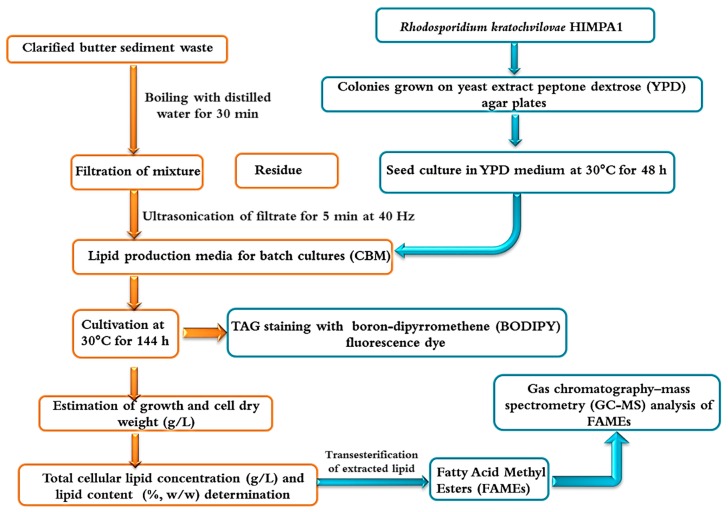
Schematic representation for the utilization of clarified butter sediment waste as a feedstock to cultivate *R. kratochvilovae* HIMPA1 for biodiesel production.

In the present study, the oleaginous yeast *Rhodosporidium kratochvilovae* HIMPA1 was used to assess the feasibility of utilizing clarified butter sediment waste as a low-cost hydrophobic substrate, particularly in comparison with conventional glucose synthetic medium. The current work aims to deliver a sustainable and environment-friendly process for the valorization of clarified butter sediment waste medium (CBM) in the production of microbial lipids that are suitable for use as biodiesel (Figure 1).

## 2. Materials and Methods

### 2.1. Materials

The chemicals and reagents used in the present study were of analytical grade and procured from Merck, Mumbai, India. Media components for yeast cultivation were purchased from Hi-Media (Mumbai, India). BODIPY_505/515_ (4,4-difluoro-1,3,5,7-tetramethyl-4-bora-3a, 4a-diaza-s-indacene) used to visualize the intracellular lipid droplets was procured from Invitrogen (Life Technology, Carlsbad, CA, USA).

### 2.2. Yeast Strain and Preparation of Seed Culture 

*R. kratochvilovae* HIMPA1 (GenBank Acc. No. KF772881) used in the present study was previously isolated by our group from permafrost soils in the Himalayan region [36]. Seed culture was prepared by inoculating a loopful of colonies from yeast extract peptone dextrose (YPD) agar plates into YPD broth (100 mL) and incubating them at 30 °C for 48 h.

### 2.3. Preparation of CBM and Yeast Cultivation

A charred (brunt) light-to-dark-brown sediment was obtained during the clarification of the collected milk cream or butter. The initial substrate of clarified butter sediment waste was milk cream. Cream (500 g) was collected from the milk after concentrating the milk fat. The cream was then heated to the boiling temperature (110–120 °C) with continuous stirring for 2–3 h, until the clear liquid could be separated from the small dark brown particles. The clarified butter was separated from solid residues by filtration through a sieve. To determine the total fat present in the solid residue, 5 g was extracted with 10 mL of *n*-hexane, in triplicates, the solvent was evaporated, and the total fat was estimated gravimetrically. The protein content was analyzed by the Kjeldahl method. To utilize the clarified butter sediment waste as a substrate for yeast cultivation, 10 g of it was boiled in 100 mL distilled water for 30 min. The mixture was filtered using a mesh strainer, and the filtrate was ultrasonicated at 40 Hz for 5 min. After cooling the filtrate, appropriate amounts of Yeast Nitrogen Base (YNB) without ammonium sulfate (0.79 g/L) and the complete supplement mixture (CSM, 1.7 g/L) were added to the mixture, whose final volume was then adjusted to 98 mL by adding distilled water. The pH of the medium was adjusted to 6.8 before autoclaving at 121 °C for 15 min. The medium was then inoculated with 2% (*v/v*) of a seed culture previously grown for 48 h, and incubated at 30 °C with 180 rpm for 144 h. Residual fat during the cultivation period was monitored by repeated *n*-hexane extraction of the supernatant. *n*-hexane was then transferred to pre-weighed glass vials and kept in the oven until solvent evaporation was complete. Finally, the residual fat (g/L) was gravimetrically determined. 

### 2.4. Determination of Cell Dry Weight and Lipid Content 

For cell dry weight determination, 10 mL of the culture was harvested every 24 h and centrifuged at 5,000 rpm. The obtained pellets were washed with distilled water, followed by washing with *n*-hexane to remove the medium components, as well as the fat present in CBM. The pellets were dried overnight on a pre-weighed aluminium boat, in an oven set at 80 °C, and the cell dry weight was gravimetrically determined. Intracellular lipids were extracted by a modified Bligh and Dyer protocol previously described by our group [36]. Total lipid concentration was estimated gravimetrically in g/L, whereas lipid content (%, w/w) was estimated using the following equation (Y) = Total lipid concentration (g/L)/cell dry weight (g/L) × 100 [36].

### 2.5. Determination of TAG Synthesis in R. kratochvilovae HIMPA1 Cells

Morphology and TAG synthesis ability of *R. kratochvilovae* HIMPA1 cultivated in CBM were observed by live fluorescence microscopy. Samples of 1 mL were harvested and washed with distilled water to remove the residual medium. The samples were washed again with *n*-hexane to remove the extra unutilized fats attached to the cells’ outer surface, which could have interfered with fluorescence microscopy. The pellets were resuspended in 50 µL distilled water and 2 µL of BODIPY_505/515_ (0.1 mg/mL in DMSO) was added to the solution in amber Eppendorf tubes to avoid photobleaching of the dye. The tubes were incubated for 5 min at room temperature. Bright field and fluorescence microscopy images were taken with an inverted fluorescence microscope (EVOS-FL, Thermo Fisher Scientific, Waltham, MA, USA) equipped with an EVOS LED light cube GFP filter (470/22 nm Excitation; 510/42 nm Emission) [37] at a 60× magnification.

### 2.6. Estimation of the Fatty Acid Profile

The extracted lipids were transesterified with acid catalysts (0.6 M HCl in methanol), as previously described [38]. The reaction was carried out in polytetrafluoroethylene (PTFE)-capped glass vials at 85 °C for 1 h and FAME were extracted with *n*-hexane after cooling the reaction mixture to the room temperature. The transesterified products were analyzed by gas chromatography-mass spectrometry (GC–MS) (Agilent, Santa Clara, CA, USA) using a DB-5MS capillary column (30 m × 0.25 mm ID and 0.25 μm film thickness). The column was initially heated at 50 °C for 1.5 min and the temperature was ramped at 25 °C/min to 180 °C, where it was maintained for 1 min. Then, the temperature was further increased to 280 °C, at a rate of 10 °C/min and maintained there for 1 min. Finally, the temperature was ramped to 250 °C at a rate of 15 °C/min and maintained at this temperature for 3 min. A total of 1 μL of the sample was loaded by autosampler in the splitless mode at 250 °C. The peaks were examined with the help of electron ionization (70 eV; scan mode 50–600 *m/z*). The temperature of the ion source was set up at 200 °C, while the mass transfer line was maintained at 250 °C. The identification of peaks was carried out by a Probability-Based Matching (PBM) library search in the MS spectral database.

### 2.7. Theoretical Analysis of the Biodiesel’s Properties 

The biodiesel’s properties were estimated by the following empirical formulae [39]:

Saponification value (SV; mg KOH) = ∑ 560 (% FC)/M
Iodine value (IV; gI_2_/100 g) = ∑ 254 DB × % FC/M
Cetane number (CN) = 46.3 + 5.458/SV − (0.255 × IV)
Degree of unsaturation (DU; % weight) = MUFA + (2 × PUFA)
Long chain saturation factor (LCSF; % wt.) = (0.1 × C16) + (0.5 × C18) + (1 × C20) + (1.5 × C22) + (2 × C24)
Cold filter plugging point ((CFPP); °C) = (3.417 × LCSF) − 16.477
Higher heating value (HHV; MJ/Kg) = 49.43 − 0.041 (SV) − 0.015 (IV)
Kinematic viscosity (ln KV at 40 °C in mm^2^/s) = −12.503 + 2.496 × ln (∑M) − 0.178 × ∑DB
Density = 0.8463 + 4.9/∑M + 0.0118 × ∑DB
Oxidative stability (OS; h) = 117.9295/(wt. % C18:2 + wt. % C18:3) + 2.5905

where M = molecular mass of each fatty acid component, DB = number of double bonds, FC = fatty acid component (%), MUFA = monounsaturated fatty acids (%), PUFA = polyunsaturated fatty acids (%).

### 2.8. Statistical Analysis

All experiments were performed in triplicates and the results were presented as the standard deviation of three independently recorded values.

## 3. Results and Discussion

### 3.1. Batch Cultivation of the Oleaginous Yeast in CBM

To reduce the cost accompanying the feedstocks for biodiesel production by oleaginous microorganisms, it is important to identify strains capable of growing on low-cost substrates [3]. While oleaginous yeasts generally prefer to utilize hydrophilic substrates such as sugars and synthesize lipids via the de novo synthesis pathway [11], some can also efficiently metabolize hydrophobic substrates via ex novo lipid synthesis [20]. In the de novo pathway, the carbon substrate is converted into the lipid precursors acetyl- and malonyl-CoA by the TCA cycle, and finally forms C14–C18 long fatty acid chains, depending on the type of microorganisms. In contrast, hydrophobic substrates are first degraded at the surface of cells in the ex novo pathway and are incorporated as such, or with modifications [40].

**Figure 2:**
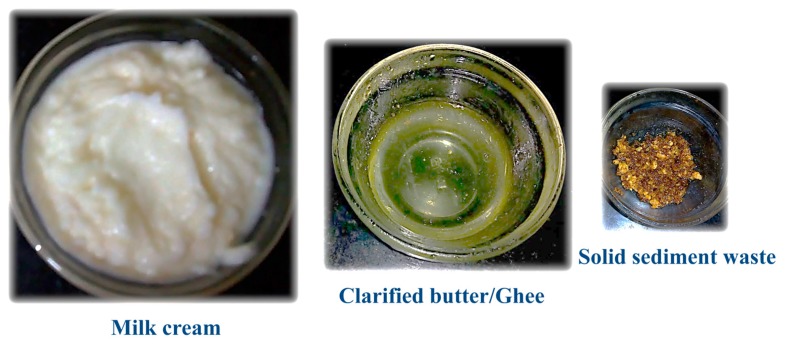
Production of clarified butter from milk cream; a charred (brunt) light-to-dark-brown solid sediment was obtained during the clarification process.

In this study, CBM was used as a hydrophobic waste substrate to cultivate *R. kratochvilovae.* A total of 500 g of milk cream was used to prepare 466 g of clarified butter or ghee (93.2% of total milk cream) and 34 g of solid residue (6.8% of total milk cream) (Figure 2). The w/w composition of the solid residue was 39.1% fat, 19.2% protein, 30.1% moisture, 2.5% lactose, and 7.3% ash. The solid residue (10 g) was boiled in 100 mL of distilled water and sonicated to facilitate fat dispersion in the medium, thus yielding 2.84% w/v of fat and 7.8 mg/mL of protein.

**Figure 3:**
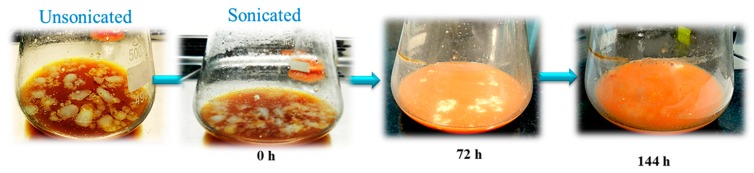
Appearance of fat from ultrasonicated clarified butter sediment waste medium (CBM) during utilization by *R. kratochvilovae* over a cultivation period of 144 h.

Hydrophobic substrates usually form unstable emulsions in aqueous solutions or float on the surface, as shown in Figure 3. To tackle this issue, oleaginous yeasts often secrete extracellular emulsifiers, such as liposan, or extracellular lipases that facilitate substrate degradation [41]. Nevertheless, to enhance the contact area between hydrophobic substrates and the cell surface, some external emulsifiers are often used to reduce the size of the hydrophobic substrates [42]. For example, in one study, oleaginous yeast *Trichosporon dermatis* CH007 was cultivated on a soybean oil-based medium along with Tween 80, Span 80, Tween 60, and OP-10 as external emulsifiers [43]. The addition of emulsifiers had a minor negative effect on the final lipid production. More importantly, though, it interfered with the quantification of residual hydrophobic substrates in the medium, due to its solubility in the organic solvent that was used for recovery of the residual hydrophobic substrates [43]. Here, CBM was ultrasonicated to reduce the fat size and make the hydrophobic substrates available in the form of easily accessible tiny droplets (Figure 3). Ultrasonication has been previously applied to facilitate the growth of *Cryptococcus curvatus* on waste cooking oil, whereby lipid accumulation was found to increase from 12.21 ± 1.34 g/L (unsonicated) to 20.34 g/L [44]. 

**Figure 4:**
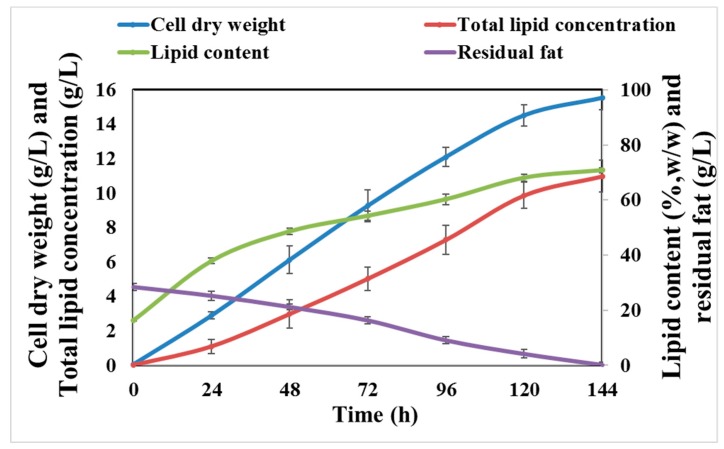
Time course showing changes in the cell dry weight, lipid content, lipid concentration, and fat consumption by *R. kratochvilovae* cultivated in CBM over a period of 144 h.

Time-course results pertaining to the cell dry weight, total lipid concentration, and lipid content for *R. kratochvilovae* cultivated in CBM are presented in Figure 4. No lag phase was observed during the initial stage of cultivation, and the cell dry weight and lipid accumulation was found to increase linearly, over a period of 144 h. The highest cell dry weight, total lipid concentration, and lipid content were 15.52 g/L, 10.98 g/L, and 70.74% (w/w), respectively after 144 h (Figure 4). Lipid content increased from 37.93% (w/w) at 24 h to 54.26% (w/w) after 72 h of cultivation. Lipid-free biomass was almost constant between 72 h and 144 h of cultivation, possibly due to simultaneous degradation and intake of fat at the cell surface. Indeed, some oleaginous yeasts, such as *Y. lipolytica*, has been shown to possess extracellular lipolytic and proteolytic activities when grown on hydrophobic substrates [20,45]. Moreover, even the filamentous fungus *Aspergillus* sp. ATHUM 3482 cultivated on a carbon-limited hydrophobic medium with waste cooking olive oil was reported to synthesize 64% (w/w) of lipids in its cellular compartment [46]. Additionally, cultivation of the oleaginous yeast *Rhodotorula glutinis* TISTR 5159 on palm oil mill effluent—another hydrophobic material—yielded 6.32 g/L of biomass, along with a 32.63% lipid content [47]. Other oleaginous yeasts and the corresponding hydrophobic waste materials that were cultivated are listed in Table 1.

Oleaginous yeasts can resort to de novo lipid synthesis when grown in the presence of hydrophilic carbon and under nitrogen-limited conditions [48]. Instead, ex novo lipid synthesis is entirely independent of nitrogen concentration when hydrophobic materials are supplied as the cultivating medium [49]. Here, cell dry weight and lipid accumIMPulation following growth of *R. kratochvilovae* HIMPA1 on the hydrophobic substrates (CBM) were considerably higher, compared to those obtained on hydrophilic substrates such as a glucose synthetic medium (GSM), containing 70 g/L of glucose and 5 g/L of ammonium sulfate [18]. The maximum cell dry weight and lipid concentration on the GSM were 13.26 g/L and 6.2 g/L, respectively, corresponding to only a 46.76% (*w/w*) lipid content [18]. During de novo lipid accumulation, oleaginous yeasts prefer to utilize glucose due to its high-energy content (~2.8 kJ/mol) [50]. However, de novo lipid accumulation is also affected by the amount of carbon source, the presence of other medium components (nitrogen, phosphorus, and sulfur), and their ratio to the carbon source (C/N, C/P, and C/S) [11,51,52]. In our previous study, the concentration of nitrogen and phosphorus was optimized so that when this oleaginous yeast was cultivated in 7% glucose with 0.1 g/L of ammonium sulfate, the highest cell dry weight and lipid concentrations were 9.23 g/L and 5.51 g/L, respectively, corresponding to 59.69% (w/cell dry weight) lipid content [18]. A similar growth pattern was observed when *Rhodosporidium kratochvilovae* SY89 was cultivated in a glucose-based medium with cane molasses as a carbon source [53]. Both values were, however, lower than the 70.74% lipid content obtained here, after 144 h of cultivation on CBM. During de novo lipid accumulation, isocitrate dehydrogenase (ICDH) is not accessible to convert isocitrate to α-ketoglutarate as ICDH is totally dependent on the concentration of AMP, and all AMP converts into inosine monophosphate, IMP by AMP-deaminase, during the condition of nitrogen starvation in the mitochondria [11]. The isocitrate accumulating in the mitochondria equilibrates with citrate via the citrate–malate–translocase system [54]. ATP–citrate lyase converts citrate to acetyl-Co-A and oxaloacetate. Finally, acetyl- Co-A and malonyl-Co-A act as lipid precursors for fatty acid chains between C14 and C16 [55]. In comparison, under similar nitrogen-limited conditions, conventional or non-oleaginous yeasts such as *Saccharomyces cerevisiae*, convert the carbon source into mannans and glucans [56]. However, the mechanisms of hydrophobic substrates assimilation by any oleaginous microorganisms are totally different from the hydrophilic substrate utilization. The assimilation of hydrophobic substrates usually starts with two different mechanisms, either by the surface-mediated transport or by direct interfacial transport [45]. After assimilation of these substrates, various pathways such as monoterminal alkane oxidation, β-oxidation, citrate, and glyoxylate cycle, work to degrade the hydrophobic substrates [21,57]. Although all of these pathways work in different compartments, such as ER, mitochondria, and peroxisome, the final destination is always β-oxidation in peroxisome [58]. The excess amount of hydrophobic substrates could be reserved in the form of lipid droplets [21].

### 3.2. Detection of TAG by Fluorescence Microscopy in R. kratochvilovae Cultivated in CBM

**Figure 5:**
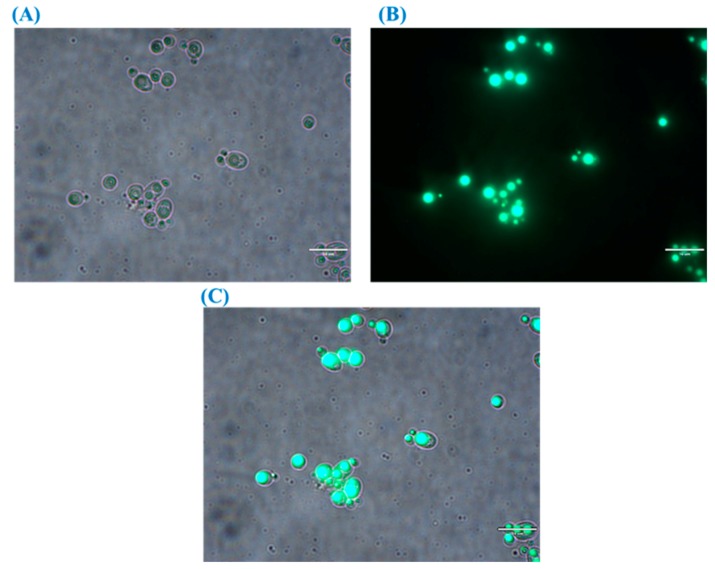
Bright field (**A**) and fluorescence (**B**) and merged images (**C**) showing lipid droplets accumulation in the oleaginous yeast *R. kratochvilovae* cultivated in CBM.

Synthesis of lipid droplets (also known as lipid bodies or adiposomes), their dynamics, size, number, and composition are totally dependent on the type of oleaginous microorganisms. Moreover, for the same strain, cultivation conditions including supplied nutrients affect the structure of lipid droplets [63]. Figure 5 shows the bright field and fluorescence images of lipid droplets in *R. kratochvilovae* HIMPA1 grown on CBM for 144 h. The image shows a direct correlation between the lipid droplets’ size and TAG accumulation under ex novo lipid synthesis. The cell and lipid droplet size of this oleaginous yeast were shown to vary with changes in the concentration of nitrogen and phosphorus in GSM [18]. Specifically, when cells were grown under nitrogen-limited conditions, lipid droplets were perfectly rounded; whereas under phosphorus-limited conditions, they were more diffuse and irregular, likely due to impaired phospholipid synthesis [18] and, thus, an aberrant monolayer surrounding the droplets was observed [57,64]. However, these variations might also reflect the different mechanisms responsible for lipid droplet synthesis. These include the ‘lensing model’ and the ‘bicelle formation model’, which describe the genesis of lipid droplets in cellular compartments [57,64]. In the lensing model, lipid droplets covered by a monolayer, originated from a single cytosolic leaflet of the endoplasmic reticulum (ER) membrane; whereas, both leaflets of the ER membrane took part in the formation of the monolayer in the bicelle model, [65]. Hence, in the case of GSM-cultivated cells, the lipid droplets were uniform and rounded; whereas, in the case of CBM-grown cells, they appeared to be more dispersed (Figure 5). 

### 3.3. Fatty Acid Profile of R. kratochvilovae Cultivated in CBM

**Figure 6:**
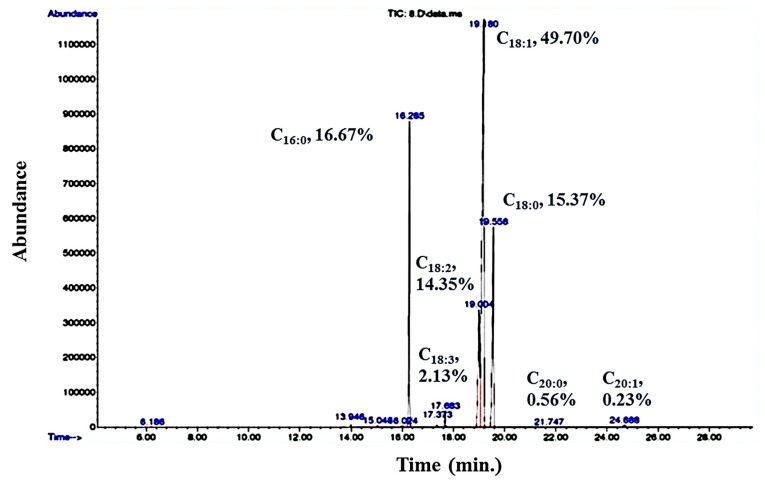
GC-MS chromatogram showing the fatty acid profile of *R. kratochvilovae* cultivated in CBM.

The lipids extracted from *R. kratochvilovae* cultivated in CBM were analyzed by GC–MS after transesterification (Figure 6). *R. kratochvilovae* HIMPA1 mainly synthesizes myristic acid (C14:0), palmitic acid (C16:0), stearic acid (C18:0), oleic acid (C18:1), along with linoleic acid (C18:2) and traces of linolenic acid (C18:3) [18]. The exact content of these fatty acids varies with the nature of the feedstock. For example, when this yeast was grown in pulp and paper industry effluent, the fatty acid content included C16:0 (21.86%), C18:0 (0.5%), C18:1 (45.43%), C18:2 (15.91%), and traces of arachidic acid (C20:0) (0.12%) [66]. In contrast, *R. kratochvilovae* HIMPA1 cultivated on hemp seed aqueous extract containing high amounts of lipids, resulted in 62.5% of saturated fatty acids (SFA), 37.5% of MUFAs, and the unusual carboceric fatty acid (C27:0) [36]. Here, a completely different profile was detected when this oleaginous yeast was cultivated in CBM; this included a high degree of unsaturation (83.09%) that comprised mainly C16:0 (16.67%), C18:0 (15.37%), C18:1 (49.70%), C18:2 (14.35%), C18:3 (2.13%), C20:0 (0.56%), and eicosenoic acid (C20:1) (0.23%) (Figure 6). In comparison, GSM-grown cultures showed a high amount of saturated fats (60.35%), including C16:0 (34.79%), C18:0 (21.32%), and C14:0 (4.24%); and fewer unsaturated ones, such as C18:1 (23.15%) and C18:2 (2.23%) [18]. 

Biodiesel quality was affected by several factors, such as fatty acids composition of the feedstock (e.g., chain length and number, and position and isomers of double-bonds), production process, refining process, and post-production parameters [67,68,69]. The properties of biodiesel obtained from the CBM- and GSM-cultivated cells are listed in Table 2, where they are also compared with the two most important international standards—the American Society of Testing and Materials ASTM 6751, and the European Standard EN 14214. 

A key parameter affecting biodiesel performance in cold environments is the CFPP, which depends strongly on the degree of unsaturation. CBM-grown cells contained a high quantity of MUFAs (48.90%), resulting in a lower CFPP (14.66 °C) than FAME obtained from the GSM-cultivated cells (31.83 °C). Oxidative stability is an essential fuel property influenced by the presence of large amounts of SFA in oil feedstock. Increased number of double-bonds or unsaturation in the fatty acids led to a higher degree of autoxidation of biodiesel and, consequently, a reduced shelf life [70]. Here, biodiesel obtained from GSM-grown cells was more stable (55.47 h) than that obtained from cells grown in CBM (9.70 h); however, both values were within the accepted international standards. In general, biodiesel has a 12% lower HHV than diesel (39.57–41.33 MJ/kg) [71]. This led to a higher consumption of biodiesel to attain a similar yield as that of conventional diesel [71,72]. Here, the biodiesel obtained from the CBM-grown cells had a slightly lower HHV (40.08 MJ/kg) than that obtained from the GSM-grown cells (42.50 MJ/kg). Kinematic viscosity determined the properties of fuel injection into the engine, with a highly viscous fuel having a larger droplet size and inferior vaporization. As shown in Table 2, both biodiesel types were well within the set international standards. In general, unsaturation in fatty acids enhanced the operability of biodiesel in a cold environment, due to a low CFPP; whereas their saturation prevented auto-oxidation of the fuel and improved the shelf life of biodiesel [73]. It is quite difficult to find a feedstock that meets all criteria for biodiesel; therefore, blending different biodiesels to achieve the optimum ratio of saturated to unsaturated fatty acids could be an effective solution [74,75].

## 4. Conclusions

Clarified sediment waste is one of the largest waste materials of the dairy industry. It is usually discarded in the surrounding environment or drained in water bodies at industrial, as well as domestic level, thus severely affecting the aquatic ecosystem and generating a putrid odor. Due to the presence of a high amount of fat, it cannot be degraded except by oleaginous microorganisms, which secrete extracellular lipases and engage in ex novo lipid synthesis. In this study, clarified sediment waste was successfully utilized as hydrophobic substrate by an oleaginous yeast, *R. kratochvilovae* HIMPA1, which synthesized 70.74% *w/w* lipids in its cellular compartment over a cultivation period of 144 h. The highest cell dry weight and total lipid concentration were 15.52 g/L and 10.98 g/L, respectively. The biodiesel produced after transesterification of the lipids extracted from these cultures satisfied international standards for biodiesel fuel. Hence, utilization of clarified sediment waste not only solves the problem of waste generation, but can be also exploited as a renewable feedstock for cost-effective biodiesel production.

## Figures and Tables

**Table 1:** Oleaginous yeasts cultivated on the hydrophobic substrates.

Oleaginous Yeast	Hydrophobic Substrate	Cell Dry Weight (g/L)	Lipid Concentration (g/L)	Lipid Content (% *w/w*)	References
*Trichosporon dermatis*	Soybean oil (20 g/L)	26.7	11.6	43.4	[43]
*Cryptococcus. curvatus*	Sonicated waste cooking oil (20 g/L)	18.62	13.06	70.13	[44]
*Rhodotorula glutinis*	Waste cooking oils + crude glycerol	25.5		46 ± 5%	[59]
*Yarrowia lipolytica*	Industrial saturated fats (stearin)	12.5	6.8	54	[60]
*Candida lipolytica* 1094	Corn oil (18 g/L)		10.1	55	[61]
*Yarrowia lipolytica* mutant strains (YlB6, YlC7, and YlE1)	Waste cooking oil (100 g/L)	10.86, 7.1, and 5.84	5.97, 4.28, and 3.91	55, 60, and 67	[62]
*Rhodosporidium kratochvilovae* HIMPA1	CBM	15.52	10.98	70.74	This Study

CBM, clarified butter sediment waste medium.

**Table 2:** Theoretical estimation of biodiesel properties from the CBM-cultivated cells.

Biodiesel Properties	CBM	GSM [18]	Standard Biodiesel Parameters	
			ASTM D6751-02	EN 14214
Degree of unsaturation	83.09	27.61	-	-
Saponification value (mg/g)	199.78	160.26	0.50 min	0.50 min
Iodine value (mgI_2_/100 g)	76.97	23.71	-	120 (max)
Cetane number	56.30	74.30	47 min	51 min
Long chain saturated factor	9.91	14.13	-	-
Cold filter plugging point	14.66	31.83	-	-
Cloud point (°C)	3.77	13.30	-	-
Pour point (°C)	−2.72	7.625	-	-
Oxidation stability (h)	9.70	55.47	3 min	6 min
Higher heating value (MJ/kg)	40.08	42.50	-	-
Kinematic viscosity (mm^2^/s)	3.96	4.52	1.9–6.0	3.5–5
Density (g/cm^3^)	0.87	0.87	-	0.86–0.90

GSM, glucose synthetic medium.
